# Hair cortisol concentrations as a putative biomarker for suicidal behavior

**DOI:** 10.1038/s41386-026-02344-y

**Published:** 2026-02-06

**Authors:** Lindsay Taraban, Emily Hone, Meilin Jia-Richards, Mary Ann Kelly, Jenna M. Lindsay, Sarah Riston, Zak Hutchinson, Thomas D. Walko, Eli Goodfriend, Brian C. Thoma, Dara Sakolsky, Kehui Chen, Antoine Douaihy, David A. Brent, Anna L. Marsland, David A. Lewis, Nadine M. Melhem

**Affiliations:** 1 Department of Psychiatry, University of Pittsburgh School of Medicine, Pittsburgh, PA USA; 2 University of Pittsburgh Medical Center, Pittsburgh, PA USA; 3 Department of Psychology, University of Pittsburgh, Pittsburgh, PA USA; 4 Department of Statistics, University of Pittsburgh, Pittsburgh, PA USA

**Keywords:** Biomarkers, Risk factors

## Abstract

Suicide is the 2nd leading cause of death for young adults in the United States, and rates are particularly high among psychiatric patients in the year following psychiatric hospitalization. However, the prediction of suicidal behavior continues to be a challenge. We examined hair cortisol concentrations (HCC)—reflecting HPA axis activity over the preceding months—as an *objective* marker of risk for suicidal behavior across the full spectrum of suicidal behavior, including death by suicide. Participants were 238 young adults across the spectrum of suicide (i.e., suicide; suicide attempt; suicidal ideation; psychiatric comparison; 57% male) and 43 individuals who died by drug overdose (56% male). Data were collected via self-report, clinical interview, medical records review, and hair sampling (i.e., 3 cm segments). Multivariate regression models were used to examine the relationship between group and HCC, controlling for covariates. HCC was significantly lower in individuals who died by suicide compared to those with a suicide attempt [Difference in EMMs (SE) = −0.64 (0.20), *p* = 0.010, *d* = 0.73], suicidal ideation [Est. Diff (SE) = −0.89 (0.20), *p* < 0.0001, *d* = 1.02], and psychiatric comparison individuals [Est. Diff (SE) = −0.74 (0.26), *p* = 0.022, *d* = 0.85]. Lower HCC may serve as an objective marker that signals risk for death by suicide among high-risk adults, which have important clinical implications for the prediction and prevention of suicide. Future studies with larger sample sizes are needed to replicate these findings and to make assaying HCC accessible for its translation into clinical practice.

## Introduction

Suicide is the 2nd leading cause of death for young adults in the United States, with rates increasing 30% over the last two decades [1]. Rates of suicide are particularly high among psychiatric patients in the year following psychiatric hospitalization [2]. However, predicting who will go on to attempt suicide is a difficult task. This is due to a variety of factors, including the dynamic nature of suicidal ideation and intent with fluctuations even over short periods of time [3], and the reliance on self-report where some patients are ambivalent about their intent [4], while others deny suicidal ideation to avoid hospitalization [5, 6]. Thus, researchers and clinicians have emphasized the importance of identifying objective, reliable biomarkers that could be applied to suicide prediction and prevention efforts [7]. Parallel to this suicide crisis, opioid-related mortality in young adults has increased by over 250% over the past 20 years [8]. Between 10 and 37% of suicides are misclassified as accidental deaths [9], which can interfere with suicide surveillance and prevention efforts. In the absence of clear corroborating evidence of suicide—e.g., a suicide note, reliable witness testimony—medical examiners will classify overdose deaths as accidental or undetermined, potentially leading to an underestimate of deaths by suicide [10]. Thus, identifying objective and reliable markers of suicidal thoughts and behaviors (STBs) can benefit not only prevention and intervention efforts, but also postmortem classification of suicide versus accidental deaths.

STBs are a complex and multifactorial phenomenon. The stress diathesis model hypothesizes an underlying vulnerability to suicidal behavior that is exacerbated by an acute stressor. Allostasis represents the processes through which biological systems adapt to ongoing stressors [11]. Chronic stress constantly challenges these biological systems, which could lead to their “wear and tear” and dysregulations. Indeed, dysregulations in the hypothalamic-pituitary-adrenal (HPA) axis, which contributes to the regulation of stress responses, has been the focus of numerous studies on STBs and as such could represent an underlying diathesis or the consequence of allostatic load or overload. Meta-analyses and systematic reviews have reported higher cortisol levels (hypercortisolism) across a variety of mental disorders, including depression [12], bipolar disorder [13], schizophrenia [14], and alcohol use disorder [15], though not all studies corroborate these findings [16, 17]. Although both HPA axis hyper- and hypo-activity are significantly associated with increased risk for STBs [18], studies tend to report *lower* cortisol levels (hypocortisolism) among suicide attempters [19–21], although this association may be age-dependent [22]. Sustained HPA axis hyperactivity resulting from chronic stress may lead to hypocortisolism [23, 24]. However, most studies measure cortisol levels in individuals with a history of suicidal behavior [19, 25, 26]. Thus, it remains unclear whether HPA axis dysregulation exists prior to or is the consequence of suicidal behavior. Hair cortisol concentrations (HCC) provide a promising and reliable method for capturing an objective retrospective assessment of cortisol levels [27, 28]. HCC in the 3-cm segment of hair closest to the scalp reflect cortisol levels over the past 3 months [28]. A recent meta-analysis examining the limited body of research (i.e., 22 studies) which has examined associations between HCC and mental disorders found that HCC is higher in the context of depression and lower in the context of PTSD [28]. We previously reported lower HCC in inpatients admitted for a suicide attempt compared to those admitted for suicidal ideation, thus reflecting lower HPA axis activity in the 3 months *preceding* an attempt [29].

The present study is the first to examine HCC across the full spectrum of STBs in young adults, including those who died by suicide. We hypothesized that HCC would be lowest among those who died by suicide, followed by suicide attempters, those with suicidal ideation, and psychiatric comparison individuals. We also compared HCC postmortem in individuals who died by suicide and a group of individuals who died by accidental overdose. We hypothesized lower HCC among individuals who died by suicide compared to those who died by overdose.

## Materials and Methods

### Sample

The PROmiSe sample comprised psychiatric inpatients (*N* = 133) aged 18–30 years who were recruited from Western Psychiatric Hospital, at the University of Pittsburgh Medical Center across the spectrum of psychopathology and suicidal ideation and behavior and a postmortem sample (*N* = 78) who died by suicide (*n* = 35) or accidental overdose (*n* = 43). Exclusion criteria for the living sample included inability to consent, chronic medical disease affecting the HPA axis and inflammatory pathways (e.g., Addison’s or Cushing’s disease), pregnancy, current antibiotic use, oral corticosteroids use in the past year, and the use of anti-inflammatories or other medications that could impact neuroendocrine or immune function (e.g., immune therapies for cancer). Postmortem hair samples were collected in collaboration with the Allegheny County Medical Examiner’s (ME) Office (Pittsburgh, PA), and only suicide deaths ruled as definite by the ME were included. The next of kin of potential suicide and overdose deaths was contacted immediately to obtain consent to collect hair samples. After the cause of death was confirmed, the next of kin was mailed a letter to determine their willingness to participate in a psychological autopsy. The University of Pittsburgh Institutional Review Board approved the study; and the Committee for Oversight of Research and Clinical Training Involving Decedents or CORID at the University of Pittsburgh approved the study for the postmortem sample.

We compared individuals across the spectrum of STBs in 170 individuals, including those who died by suicide (*N* = 35) and the 135 living psychiatric inpatients distributed as 61 admitted for a suicide attempt (SA); 53 individuals who had suicidal ideation (SI); and 19 psychiatric comparison individuals (PC) without current ideation or attempt. Individuals in the SI and PC groups could have a previous history of suicide attempt, but not an attempt that occurred within the last three months (i.e., not overlapping with HCC collection). Prior history of suicidal behavior is one of the most important predictors of future suicidal behavior and as such, we included SI and PC with such history to better represent the psychiatric population at risk for suicidal behavior. In this sample, 21% percent of the 19 PC (*n* = 4) had a prior suicide attempt, with a mean of 2.42 years since the last attempt [range = 162–1736 days; SD = 772.52 days] and twenty eight percent (*n* = 15) of the 53 SI participants had a prior suicide attempt, with a mean of 1.41 years since the last attempt [range = 105–5100 days, SD = 1327.56 days]. We also conducted a mega-analysis, which included an additional 70 participants (35 SA and 35 SI) from a pilot sample who were aged 18–30, had HCC data available, and were recruited from the same source and had similar inclusion and exclusion criteria as the living sample described above [30]. The total sample sizes included in the mega-analyses were 35 suicide deaths, 96 SA, 88 SI, and 19 PC (*N* = 238). Group composition of each sample is reported in Supplementary Table 1. Comparisons between the PROmiSe and pilot samples are displayed in Supplementary Table 2.

### Assessments

Participants from the PROmiSe or “Promising biomarkers for suicidal behavior in youth” Study sample reported on lifetime and current suicidal ideation and behavior and lethality of attempts via structured interview using the *Columbia-Suicide Severity Rating Scale* (C-SSRS) [30]. The frequency of suicidal ideation was assessed using the *Adult Suicidal Ideation Questionnaire* (ASIQ) [31]. We administered the *Structured Clinical Interview for DSM-5 Diagnoses* (SCID-5) [32] to obtain lifetime and current psychiatric history. Masters- and PhD-level clinicians with experience in psychological assessment, psychological autopsy, and the assessment of suicidal ideation and behavior administered these interviews. All interviews were presented in diagnostic conferences using best-estimate procedures integrating all sources of data, overseen by an experienced clinician (co-author D.S.). We also obtained consent to access medical records for information about medical diagnoses, treatment, and medications. Participants also reported on deaths by suicide among first-degree relatives (Self-Injurious Thoughts and Behaviors Interview) [33]. Height and weight were measured to compute body mass index (BMI). Participants from the pilot sample used in the mega-analysis reported on similar domains of psychopathology, suicidality, and demographics (see Supplemental Table 2).

For the postmortem sample, the next of kin completed a structured psychological autopsy that assessed medical and psychiatric history, family history, and medication and tobacco use at the time of death. Lifetime history of psychiatric diagnoses, including diagnoses at the time of death, were assessed using the *SCID-5*, with consensus diagnoses made in consultation with three experienced clinicians not involved in the study. Prior history of suicide attempts and possible factors contributing to attempts were assessed using the SITBI [33]. Global functioning at the time of death was assessed using the *Strauss-Carpenter Outcome Scale* [34]. BMI was obtained from the ME.

### Hair assays

HCC was determined by collecting the 3 cm of hair closest to the scalp (~50 mg of hair) to determine system cortisol production over the past 3 months. Hair samples were stored at room temperature in the dark prior to processing. Methods for processing the hair samples included a step to wash hair in isopropanol alcohol for 3 min to remove any hair products/dyes and to remove biological debris (e.g., blood) that could influence results [35]. Hair was pulverized, and cortisol was extracted following methods described by Laudenslager et al. [36]. Cortisol was quantified by enzyme immunoassay run using manufacturer’s instructions (High sensitivity salivary cortisol EIA # 3002; Salimetrics, LLC, PA). Samples were run in duplicate, with an average coefficient of variation between duplicates of 4.9% for the living sample and 4.2% for the postmortem sample. Technicians were blind to group status. We examined the effect of hair bleaching on HCC in the living sample and found that those who bleached their hair had similar levels of HCC compared to those who did not [*M*_bleached_ = 2.38, SD_bleached_ = 0.93; *M*_unbleached_ = 2.42, SD_unbleached_ = 0.94; t(7,72) = −0.06, *p* = 0.951, *d* = 0.03]. We also examined the time interval between suicide attempt and hair sample collection among the attempters. The average number of days was 11 (SD = 9 days), ranging from 1 to 38 days. Interval times between the date of the attempt and hair sample collection were not associated with HCC (*β* = 0.06, SE = 0.19, *p* = 0.744), controlling for batch and BMI. In the postmortem sample, the average postmortem interval (PMI) between death and hair samples collected was 22 days (SD = 27 days, range from 5 to 112 days). PMI was not significantly associated with HCC (*β* = 0.11, SE = 0.12, *p* = 0.318) even after controlling for batch and BMI. Identical methods were used for processing hair from the PROmiSe and pilot samples. All samples were stored identically.

### Statistical analyses

Raw HCC values were positively skewed and thus, we conducted a natural logarithmic transformation, which resulted in a normal distribution of HCC (Shapiro-Wilk normality test = 0.99, *p* = 0.156; Supplemental Fig. 1). All analyses were conducted in R (Version 4.4.1) [37]. For all our analyses, we first compared groups on demographic characteristics, clinical characteristics, and HCC levels using t-tests, analysis of variance (ANOVA), Pearson’s chi-squared, and Fisher’s exact tests. Post-hoc pairwise comparisons were calculated using estimated marginal means with Tukey-adjusted p values. We then examined the relationship of HCC to demographic and clinical characteristics, using linear regression and controlling for HCC’s assay batch, to identify covariates associated with HCC at the *p* < 0.10 level. Ordinal logistic regression was used to examine the relationship between HCC and the severity of suicidal behaviors across the full spectrum, and the relationship between HCC and the lethality of a suicide attempt. Lethality was coded using the C-SSRS from 0 to 5 where 0 = no or very minor physical damage and 5 = death. To examine the relationship of HCC to suicidal behavior across the full spectrum while controlling for covariates, we ran multivariate regression models. We conducted hierarchical modeling introducing first demographics (sex, race, age, and BMI), regardless of whether they were significantly associated with HCC or not at the univariate level. BMI was included as a covariate based on evidence of a positive relationship between BMI and HCC [38]. We then introduced clinical covariates associated with HCC at *p* < 0.10 at the univariate level. Clinical covariates were similar in nature and timeframe and as such we did not separate them into additional categories. Finally, to obtain the most parsimonious model, we removed variables that were not significant at *p* < 0.05. We assessed interactions between group and covariates in the final models. None of these interactions were significant and thus are not discussed further. All models adjusted for HCC assay batch regardless of significance. We also conducted sensitivity analyses excluding SI and PC with prior history of suicidal behavior. Cohen’s *d*, odds ratios, or R [2] are presented where appropriate as measures of effect size. These analyses were not officially preregistered but were consistent with the primary aims and analyses detailed in the PROmiSe R01 grant application (MH109493).

## Results

### Full spectrum of STBs

#### PROmiSe sample

**Fig. 1:**
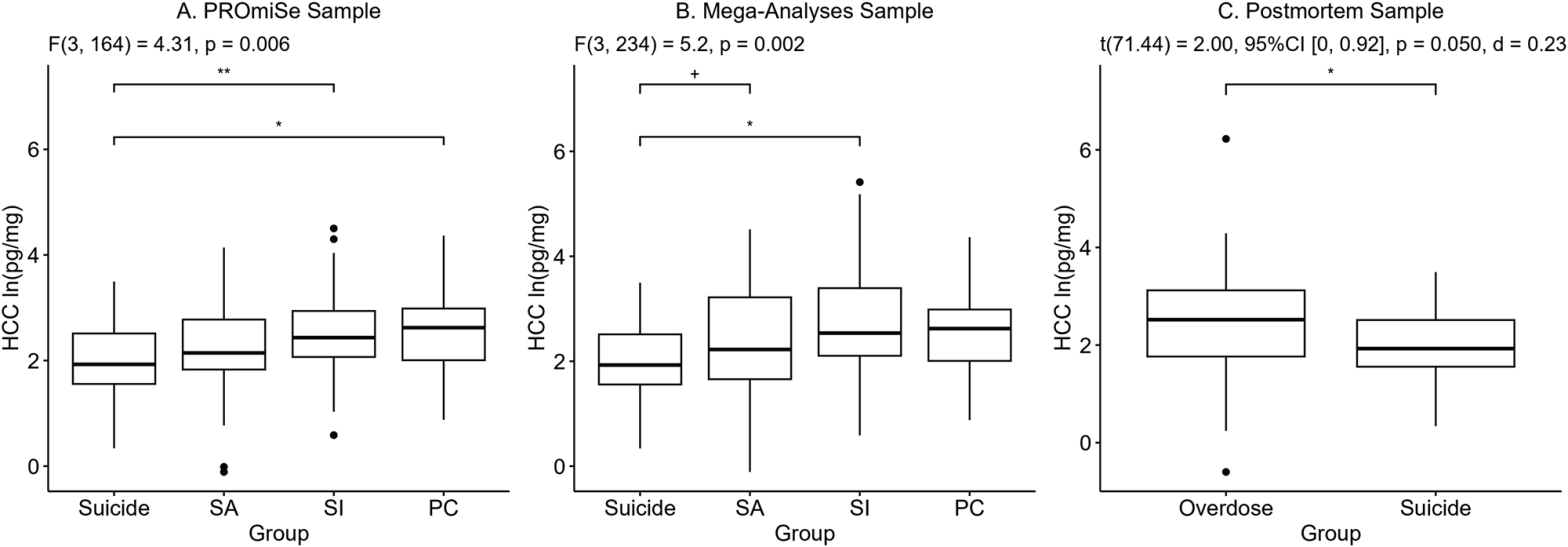
Boxplots showing hair cortisol concentration (HCC) comparisons between groups for the PROmiSe, Mega-Analyses, and Postmortem samples. PC psychiatric control group, SI suicidal ideation group, SA suicide attempt group. Corresponding tables with group contrast effect sizes for *A* and *B* plots can be found in Tables S5 and S15, respectively.

Demographic and clinical comparisons of those who died by suicide, SA, SI, and PC are reported in Supplemental Table 3. Individuals who died by suicide were significantly older, and more likely to be taking antidepressants and psychotropics compared to all other groups; they were more likely to be male and have a lifetime history of depression compared to those in the SA and SI groups and had lower rates of PTSD compared to the SI group. We also examined associations of HCC with demographic and clinical characteristics (Supplemental Table 4). We found significant group differences in HCC [F(3, 166) = 4.38, *p* = 0.005; Fig. 1A], where pairwise comparisons indicated HCC was significantly lower in those who died by suicide compared to those with SI [Difference in EMMs (SE) = −0.57(0.18), *p* = 0.010, *d* = 0.69] and PC [Difference in EMMS (SE) = −0.63(0.24), *p* = 0.042, *d* = 0.76; Supplemental Table 5]. The difference between those who died by suicide compared to those with SA was not significant [Difference in EMMs (SE) = −0.25(0.17), *p* = 0.481, *d* = 0.30]. We also assessed whether there was a linear relationship between HCC and suicidal behavior and found that every unit decrease in HCC was associated with increased odds of higher severity on the spectrum of SB [OR = 0.56, 95% CI = (0.40, 0.78), *p* = 0.001, *d* = 0.32]. Similarly, we found a significant association between HCC and the lethality of suicidal behavior whereby every unit decrease in HCC was associated with increased odds of a higher lethality rating for suicide attempts [OR = 0.65, 95% CI = (0.44, 0.94), *p* = 0.022, *d* = 0.24].

To examine the association of HCC with suicidal behavior while controlling for covariates, we conducted multivariable modeling and included covariates significantly associated with HCC. Group continued to be significantly associated with HCC [*F*(3, 158) = 5.41, *p* = <0.001; Table 1], with pairwise comparisons showing lower HCC in those who died by suicide compared to SI [Difference in EMMs (SE) = −0.66 (0.18), *p* = 0.002] and PC [Difference in EMMs (SE) = −0.61 (0.22), *p* = 0.033, Table 2], with large effect sizes (*d* = 0.87 and 0.80 respectively). A medium effect size was found between those who died by suicide and SA [*d* (95% CI) = 0.44 (−0.05, 0.93)] that did not reach statistical significance, with lower HCC among those who died by suicide versus SA (Table 2). Our final model included sex and HCC batch. We conducted sensitivity analyses by removing influential points, removing (*n* = 5) participants whose HCC assays had coefficient of variation (CV) over 15%, and by removing any PC (*n* = 4) and SI (*n* = 19) participants with any history of suicide attempt. Similar results were obtained (Supplemental Tables 6–11). Given that SUD medications were associated with HCC, we also conducted sensitivity analyses by removing participants taking SUD medication (*n* = 5) and obtained similar results (Supplemental Tables 12 and 13).

**Table 1:** Final multivariate models predicting HCC in the PROmiSe and mega-analyses samples.

PROmiSe Sample					Mega-Analyses Sample				
Variable	Beta	*SE*	*t*	*p*	Variable	Beta	*SE*	*t*	*p*
Intercept	1.94	0.21	9.39	<0.001***	Intercept	2.76	0.29	9.6	<0.001***
Group (Ref = PC)			F(3, 158) = 5.41	0.001**	Group (Ref = PC)			F(3, 222) = 7	<0.001***
SI	0.05	0.21	0.24	0.811	SI	0.14	0.23	0.61	0.545
SA	−0.27	0.21	−1.31	0.191	SA	−0.10	0.24	−0.44	0.661
Suicide	−0.61	0.22	−2.76	0.006**	Suicide	−0.74	0.26	−2.88	0.004**
Sex (Male)	0.46	0.12	3.66	<0.001***	Sex (Male)	0.29	0.12	2.33	0.021*
Batch			F(5, 158) = 4.71	<0.001***	Bipolar (lifetime)	0.33	0.15	2.27	0.024*
					SUD (lifetime)	0.22	0.12	1.81	0.072
					Sample (PROmiSe)	−0.60	0.15	−3.99	<0.001***
					Batch			F(5, 222) = 8.25	<0.001***

PROmiSe sample: *N* = 170, *F*(9, 160) = 5.83, *R*^2^ = 0.25, *p* < 0.001. Mega-analyses sample: *N* = 237, *F*(12, 224) = 5.53, *R*^2^ = 0.29, *p* < 0.001.

*HCC* hair cortisol concentration ln(pg/mg), *SI* suicidal ideation group, *SA* suicide attempt group, *SUD* substance use disorder.

*p < 0.05; **p < 0.01; ***p < 0.001.

**Table 2:** Post-hoc pairwise comparisons results for final multivariate models predicting HCC.

Contrast	PROmiSe Sample			Mega-Analyses Sample		
	Est. Diff (*SE*)	*p*	*d*(95% CI)	Est. Diff (*SE*)	*p*	*d*(95% CI)
SA - PC	−0.27(0.21)	0.556	0.36(−0.18, 0.91)	−0.10 (0.24)	0.972	0.12 (−0.42, 0.66)
SI - PC	0.05(0.21)	0.995	0.07(−0.47, 0.60)	0.14 (0.23)	0.930	0.16 (−0.37, 0.70)
SI - SA	0.32(0.15)	0.123	0.43(0.04, 0.81)	0.25 (0.13)	0.247	0.28 (−0.02, 0.58)
Suicide - PC	−0.61(0.22)	0.033*	0.80(0.22, 1.38)	−0.74 (0.26)	0.022*	0.85 (0.26, 1.44)
Suicide - SA	−0.33(0.19)	0.292	0.44(−0.05, 0.93)	−0.64 (0.20)	0.010**	0.73 (0.27, 1.20)
Suicide - SI	−0.66(0.18)	0.002**	0.87(0.39, 1.35)	−0.89 (0.20)	<0.001***	1.02 (0.56, 1.48)

Differences calculated using estimated marginal means with Tukey-adjusted *p* values.

*HCC* hair cortisol concentration ln(pg/mg), *PC* psychiatric control group, *SI* suicidal ideation group, *SA* suicide attempt group.

*p < 0.05, **p < 0.01, ***p < 0.001.

#### Mega-analytic sample

Overall, results from the mega-analysis followed a similar pattern to those of the PROmiSe sample, though with larger effect sizes (Supplemental Tables 14–16). We found significant group differences in HCC [*F*(3, 236) = 5.27, *p* = 0.002; Fig. 1B], where pairwise comparisons indicated HCC was significantly lower in those who died by suicide compared to those with SI [Difference in EMMs (SE) = −0.75(0.19), *p* = 0.001, *d* = 0.77; Supplemental Table 16]. Similar to our results for the PROmiSe sample, we found that every unit decrease in HCC was significantly associated with increased odds of higher severity on the spectrum of SB [OR = 0.66, 95% CI = (0.52, 0.84), *p* = 0.001, *d* = 0.23].

Controlling for covariates, we again found significant group differences in HCC [*F*(3, 224) = 7.10, *p* < 0.0001; Table 1], and pairwise comparisons indicated HCC was significantly lower in those who died by suicide compared to those with SA [Difference in EMMs (SE) = −0.64 (0.20), *p* = 0.009, *d* = 0.74], SI [Difference in EMMs (SE) = −0.88 (0.20), *p* = 0.0001, *d* = 1.02], and PC [Difference in EMMs (SE) = −0.74 (0.26), *p* = 0.021, *d* = 0.86; Table 2]. We conducted sensitivity analyses removing influential points, those with HCC assays with CVs over 15%, those on SUD medications, and PC and SI participants with a history of suicide attempt and obtained similar results (Supplementary Tables 6–11).

### Postmortem sample: suicide versus overdose

Demographic and clinical comparisons of those who died by suicide versus overdose are reported in Supplementary Table 17. The associations of HCC with demographic and clinical characteristics are reported in Supplementaryl Table 18. We found that those who died by suicide showed lower HCC compared to those who died by overdose [Welch’s *t*(71.44) = 2.00, *p* = .050, *d* = .23; Fig. 1C]. Controlling for covariates, the final multivariable model included batch and lifetime seizure disorder as covariates and the difference between those who died by suicide and overdose deaths was no longer significant [${{{\rm{\beta }}}}({{{\rm{SE}}}})$ = −0.38(.22), *p* = 0.087; Table 3]. In this model, the effect size between the two groups was a medium effect size [*d* = 0.4, CI = (−0.06, 0.87)]. These results remained consistent after sensitivity analysis removing influential points and removing participants with CVs > 15% (Supplementay Tables 19 and 20). We also examined the postmortem interval (PMI) between death and the collection of hair samples and identified one outlier with a PMI of 112 days. We conducted sensitivity analyses removing this outlier and our results were unchanged (Supplementary Table 21). None of the postmortem participants were taking SUD medications.

**Table 3:** Final multivariate regression model predicting HCC in the postmortem sample.

Variable	Beta	*SE*	*t*	*p*
Intercept	1.89	0.18	10.56	<0.001 ***
Group: Suicide	−0.38	0.22	−1.74	0.087
Seizure Disorder (lifetime)	1.00	0.36	2.75	0.007 **
Batch			*F*(2, 72) = 8.21	0.001 **

*N* = 77, *F*(4, 72) = 7.39, *R*^2^ = 0.29, *p* < 0.001. Suicide-Overdose contrast effect size: *d* = 0.4, 95% CI [−0.06, 0.87].

*HCC* hair cortisol concentration ln(pg/mg), *Seizure Disorder* lifetime history of seizure, including psychogenic nonepileptic seizures.

*p < 0.05; **p < 0.01; ***p < 0.001.

## Discussion

We found HCC to be significantly lower in those who died by suicide compared to those with suicide attempts (SA), suicidal ideation (SI), and psychiatric comparison individuals (PC), with large effect sizes.

We discuss these findings in the context of the strengths and limitations of this study. This is the first study to use HCC to compare cortisol levels across the full spectrum of STBs, including those who died by suicide. Although our effect sizes were medium-to-large, some of the pairwise comparisons fell short of statistical significance within the PROmiSe Study sample. However, adding participants in the SA and SI groups in the mega-analysis resulted in increased power to detect these medium to large effect sizes as statistically significant. While the number of PC individuals (with no current suicidal ideation or behavior) was small, we were still able to detect significant differences in HCC between this group and those who died from suicide. For the HCC comparison between suicide deaths and those who died by overdose, we found a similar direction whereby HCC was lower, although not statistically significant after controlling for covariates, among those who died from suicide versus those classified as dying from overdose with a medium effect size. Our sample size was small to detect this effect size; however, this was a very stringent comparison given prior research indicating a misclassification bias whereby some accidental overdoses may have been intentional suicidal behavior but lack data (e.g., such as a suicide note indicating intent) to classify them as such [9, 39]. Chronic opioid use has been associated with lower levels of HCC [40], further increasing the difficulty of finding distinct HCC profiles between these groups. Although the pattern of our results was consistent with other findings in this manuscript (i.e., lower HCC for completed suicide versus accidental overdose), our results contrast with a prior study which found *higher* levels of HCC among both individuals with depression (*n* = 20) and those who had died by suicide (*n* = 45) compared to a control group of females (*n* = 22) without any history of mental disorders [41]. Thus, additional research with larger samples are needed to better understand the relationship between HCC and STBs. Finally, we used a 3 cm segment of hair to measure HCC, reflecting cortisol levels across the three months prior to suicidal behavior. Future studies are needed to examine smaller 1-cm segments [28, 41], which would allow for more precise estimation of cortisol levels in the month preceding STBs and thus its role in acute risk.

Prior research has consistently shown HPA axis dysregulation across psychiatric disorders, but results have been mixed with respect to the direction of findings [12, 14]. For example, cortisol—including HCC—tends to be higher in the context of depression [12, 28], and lower in the context of PTSD [28]. Differences across studies may be due to methodological differences in the assessment of cortisol (e.g., salivary, blood cortisol), or underlying differences in diagnoses, associated risk factors, or other characteristics of the patient population. We found a significant and consistent pattern of lower HCC being associated with increased risk for suicidal behavior. HCC has the advantages of capturing cortisol levels retrospectively, being easily accessible, and being less affected by time of day and environmental factors, as is the case with salivary cortisol [28]. We found HCC to be significantly increased in males across the spectrum of risk for suicidal behavior; however, we found no significant group by sex interaction. This could be due to our limited power to detect such interaction. Males are at disproportionately increased risk for death by suicide compared to females with 4:1 ratio, respectively; while females are 1.5–2 times two times at higher risk to attempt suicide [42–44]. Future studies with larger samples are needed to better understand sex differences in HCC and risk for suicidal behavior.

Our results are an important contribution showing hypocortisolism preceding death by suicide. These results are consistent with our previous study where we found lower HCC in a group of SA compared to SI and healthy controls [29]. These results are also consistent with prior work—including our own [20, 21, 45]—reporting basal cortisol levels to be lower among suicide attempters compared to other high risk participants prior to engaging in a stressful social task [19, 46]. Specifically, hypercortisolism may result from sustained HPA axis hyperactivity due to chronic stress through the suppression of CRH and ATCH secretion driven by chronically elevated cortisol. This could result with a diminished ability to mount an adaptive stress response [23, 47], consistent with the construct of allostatic load or overload [11], which may put individuals at increased risk for STBs when faced with an acute stressor. In addition, the HPA axis has tight signaling with inflammatory pathways whereby cortisol has anti-inflammatory properties through its regulation of the transcription of proinflammatory cytokine genes. Dysregulations in HPA axis activity, whether hypo- or hypercortisolism, unleashes its control on inflammatory pathways activating the kynurenine pathways and neuroinflammatory processes that in turn affect serotonergic activity [48]. Indeed, studies have previously reported lower cortisol levels prior to an experimental stressor to be associated with higher binding to the serotonin 1 A receptor using [11 C]WAY-100635 [49]. Lower basal cortisol as measured in saliva and plasma have also been associated with greater impulsivity [50, 51] and history of childhood trauma [52, 53], while higher levels of salivary cortisol and acute cortisol administration have been associated with impaired executive functioning [54, 55]. Impulsivity, history of childhood trauma, and impaired executive functioning are major risk factors for suicidality [53, 56, 57]. Future studies are needed to better understand the mechanisms through which HPA axis dysregulation increases risk for suicidal behavior.

In conclusion, HCC may serve as an objective marker that signals risk for suicide among high-risk individuals. Our findings have important clinical implications for the identification and prevention of suicidal behavior. Future studies with larger sample sizes are needed to replicate these findings and to make assaying HCC accessible for its translation into clinical practice.

## Supplementary information

Supplementary Materials

## Data Availability

Data used for this study are not publicly available but may be shared upon request to the corresponding author.
