# Supplementary material for: Hair cortisol concentrations as a putative biomarker for suicidal behavior
Source: Neuropsychopharmacology. 2026 Feb 6;51(6):1084–90. doi: 10.1038/s41386-026-02344-y (PMC13125511; doi:10.1038/s41386-026-02344-y)
Supplement: Supplementary file 1 — Supplementary Materials [file 41386_2026_2344_MOESM1_ESM.docx]

**Supplementary Materials**

**Table of Contents**

[Table 1. Group Composition of Each Sample 2](#_Toc216956225)

[Table 2. Sample Comparisons for Mega-Analyses Sample 3](#_Toc216956226)

[Table 3. Sample Characteristics and Group Comparisons Across the Spectrum of Suicidal Behaviors in the PROmiSe Sample 4](#_Toc216956227)

[Table 4. Relationships of Demographic and Clinical Characteristics to HCC Across the Spectrum of Suicidal Behaviors in the PROmiSe Sample 5](#_Toc216956228)

[Table 5. Pairwise Comparisons for HCC in PROmiSe Sample 5](#_Toc216956229)

[Table 6. Sensitivity Analysis Removing Influential Points in PROmiSe and Mega-Analyses Samples 6](#_Toc216956230)

[Table 7. Pairwise Comparisons for Sensitivity Model in the PROmiSe and Mega-Analyses Samples with Influential Points Removed 6](#_Toc216956231)

[Table 8. Sensitivity Analyses for the PROmiSe and Mega-Analyses Samples—Removing Participants with CV > 15% (*n* = 5) 7](#_Toc216956232)

[Table 9. Pairwise Comparisons for PROmiSe and Mega-Analyses Final Multivariate Models Removing Participants with CVs > 15% (*n* = 5) 7](#_Toc216956233)

[Table 10. Sensitivity Analyses for the PROmiSe and Mega-Analyses Samples—PC (*n* = 4) and SI (*n* = 19) Participants with History of Suicide Attempt Removed 8](#_Toc216956234)

[Table 11. Pairwise Comparisons for PROmiSe and Mega-Analyses Final Multivariate Models Removing PC (*n =* 4) and SI (*n* = 19) Participants with History of Suicide Attempt 8](#_Toc216956235)

[Table 12. Final Multivariate Regression Models for the PROmiSe and Mega-Analyses Samples—Participants Taking SUD Medications Removed (*n* = 5) 9](#_Toc216956236)

[Table 13. Post-Hoc Pairwise Comparisons in Final Models for the PROmiSe and Mega-Analyses Samples with Participants Taking SUD Medications Removed (*n* = 5) 9](#_Toc216956237)

[Table 14. Sample Characteristics and Group Comparisons in Mega-Analysis Sample 10](#_Toc216956238)

[Table 15. Relationships of Demographic and Clinical Characteristics to HCC in Mega-Analyses Sample 11](#_Toc216956239)

[Table 16. Pairwise Comparisons for HCC in Mega-Analyses Sample 11](#_Toc216956240)

[Table 17. Sample Characteristics and Group Comparisons in the Postmortem Sample 12](#_Toc216956241)

[Table 18. Relationships of Demographic and Clinical Characteristics to HCC in the Postmortem Sample 14](#_Toc216956242)

[Table 19. Sensitivity Analysis in the Postmortem Sample with Influential Points Removed (*n* = 3) 15](#_Toc216956243)

[Table 20. Sensitivity Analyses for the Postmortem Sample—Removing Participants with CV > 15% (*n* = 1) 15](#_Toc216956244)

[Table 21. Sensitivity Analyses for the Postmortem Sample—Removing Participant with Long Postmortem Interval Time 15](#_Toc216956245)

[Figure 1. Distribution of log-transformed hair cortisol concentration data 16](#_Toc216956246)

# Table 1. Group Composition of Each Sample

| **Group** | **Overall** *N* = 281 | **Postmortem** *n* = 78 | **PROmiSe** *n* = 133 | **Pilot** *n* = 70 |
| --- | --- | --- | --- | --- |
| PC | 19(6.8%) | -- | 19(14%) | 0(0%) |
| SI | 88(31%) | -- | 53(40%) | 35(50%) |
| SA | 96(34%) | -- | 61(46%) | 35(50%) |
| Suicide | 35(12%) | 35(45%) | -- | -- |
| Overdose | 43(15%) | 43(55%) | -- | -- |
| *Note*. PC = psychiatric control group; SI = suicide attempt group; SA = suicide attempt group. | | | | |

# Table 2. Sample Comparisons for Mega-Analyses Sample

|  |  | **Mean(*SD*) or *n*(%)** | | |  |  |  |  |
| --- | --- | --- | --- | --- | --- | --- | --- | --- |
|  | ***N*** | **Overall**  *N* = 240 | **PROmiSe**  *n* = 170 | **Pilot**  *n* = 70 | **Test** | **Statistic** | ***p*** | ***d*(95% CI)** |
| **Participant Characteristics** | | | | | | | | |
| Group | 238 |  |  |  | Χ²(3) | 29.35 | <0.001*** |  |
| PC |  | 19(8.0%) | 19(11%) | 0(0%) | Χ²(1) | 8.60 | 0.003** | 0.42(0.14, 0.70) |
| SI |  | 96(40%) | 61(36%) | 35(50%) | Χ²(1) | 3.85 | 0.050* | 0.28(0.00, 0.56) |
| SA |  | 88(37%) | 53(32%) | 35(50%) | Χ²(1) | 7.22 | 0.007** | 0.39(0.11, 0.67) |
| Suicide |  | 35(15%) | 35(21%) | 0(0%) | Χ²(1) | 17.10 | <0.001*** | 0.61(0.32, 0.89) |
| Age | 238 | 24.25(4.68) | 24.66(4.94) | 23.24(3.82) | t(237) | 2.39 | 0.018* | 0.31(0.03, 0.59) |
| BMI | 213 | 25.37(5.51) | 25.73(5.87) | 24.63(4.65) | t(212) | 1.48 | 0.141 | 0.20(−0.09, 0.49) |
| Sex (Male) | 238 | 135(57%) | 90(54%) | 45(64%) | Χ²(1) | 2.31 | 0.128 | 0.22(−0.06, 0.50) |
| Race (White) | 237 | 189(80%) | 132(79%) | 57(81%) | Χ²(1) | 0.17 | 0.677 | 0.06(−0.22, 0.34) |
| **Lifetime Psychiatric Diagnoses** | | | | | | | | |
| Unipolar Depression | 235 | 165(70%) | 125(76%) | 40(57%) | Χ²(1) | 8.14 | 0.004** | 0.41(0.13, 0.69) |
| Bipolar Disorder | 235 | 49(21%) | 23(14%) | 26(37%) | Χ²(1) | 16.03 | <0.001*** | 0.59(0.30, 0.87) |
| Anxiety Disorders | 235 | 102(43%) | 55(33%) | 47(67%) | Χ²(1) | 22.87 | <0.001*** | 0.71(0.43, 1.00) |
| Psychosis | 235 | 7(3.0%) | 5(3.0%) | 2(2.9%) | Fisher's | -- | 1.000 | 0.01(−0.27, 0.29) |
| PTSD | 235 | 90(38%) | 33(20%) | 57(81%) | Χ²(1) | 78.48 | <0.001*** | 1.54(1.23, 1.85) |
| AUD | 235 | 104(44%) | 65(39%) | 39(56%) | Χ²(1) | 5.31 | 0.021* | 0.33(0.05, 0.61) |
| SUD | 235 | 143(61%) | 93(56%) | 50(71%) | Χ²(1) | 4.68 | 0.030* | 0.31(0.03, 0.59) |
| **Current Medication Use** | | | | | | | | |
| Psychotropics | 158 | 146(92%) | 108(92%) | 38(93%) | Fisher's | -- | 1.000 | 0.01(−0.34, 0.37) |
| Antidepressants | 161 | 124(77%) | 93(78%) | 31(76%) | Χ²(1) | 0.06 | 0.804 | 0.04(−0.31, 0.40) |
| Antipsychotics | 161 | 41(25%) | 34(28%) | 7(17%) | Χ²(1) | 2.04 | 0.153 | 0.26(−0.10, 0.61) |
| Anticonvulsant | 161 | 27(17%) | 23(19%) | 4(9.8%) | Χ²(1) | 1.94 | 0.164 | 0.25(−0.10, 0.61) |
| Antianxiety | 161 | 60(37%) | 48(40%) | 12(29%) | Χ²(1) | 1.51 | 0.220 | 0.22(−0.13, 0.58) |
| SUD Medications | 161 | 8(5.0%) | 5(4.2%) | 3(7.3%) | Fisher's | -- | 0.421 | 0.14(−0.21, 0.50) |
| Birth Control | 112 | 7(6.3%) | 2(2.8%) | 5(12%) | Fisher's | -- | 0.098 | 0.39(0.00, 0.78) |
| **Outcome** | | | | | | | | |
| Hair Cortisol Concentration ln(pg/mg) | 238 | 2.46(1.00) | 2.29(0.85) | 2.87(1.20) | t(237) | −3.69 | <0.001*** | 0.60(0.32, 0.89) |
| *Note.* *p<0.05; **p<0.01; ***p<0.001. BMI = body mass index; PTSD = posttraumatic stress disorder; AUD = alcohol use disorder; SUD = substance use disorder. PC = psychiatric control group; SI = suicidal ideation group; SA = suicide attempt group; BMI = body mass index; PTSD = posttraumatic stress disorder; AUD = alcohol use disorder; SUD = substance use disorder. | | | | | | | | |

# Table 3. Sample Characteristics and Group Comparisons Across the Spectrum of Suicidal Behaviors in the PROmiSe Sample

|  |  | **Mean(*SD*) or *n*(%)** | | | | |  |  |  |
| --- | --- | --- | --- | --- | --- | --- | --- | --- | --- |
|  | ***N*** | **Overall**  *N* = 168 | **PC**  *n* = 19 | **SI**  *n* = 53 | **SA**  *n* = 61 | **Suicide**  *n* = 35 | **Test** | **Statistic** | ***p*** |
| **Sample Characteristics** | | | | | | | | | |
| Age | 168 | 24.66(4.94) | 23.04(3.46)^b^ | 24.55(4.08)^b^ | 23.70(3.96)^b^ | 27.40(7.03)^a^ | F(3, 164) | 3.51 | 0.020* |
| BMI | 168 | 24.66(4.94) | 23.04(3.46)^b^ | 24.55(4.08)^b^ | 23.70(3.96)^b^ | 27.40(7.03)^a^ | F(3, 164) | 3.51 | 0.020* |
| Sex (Male) | 168 | 90(54%) | 13(68%)^ab^ | 26(49%)^b^ | 24(39%)^b^ | 27(77%)^a^ | Χ²(3) | 14.90 | 0.002** |
| Race (White) | 167 | 132(79%) | 14(74%)^ab^ | 42(79%)^ab^ | 43(72%)^b^ | 33(94%)^a^ | Fisher's | -- | 0.045* |
| Current Tobacco Use | 136 | 61(45%) | 8(50%) | 22(51%) | 21(41%) | 10(38%) | Χ²(3) | 1.57 | 0.666 |
| Education | 143 |  |  |  |  |  | Fisher's | -- | 0.653 |
| < High School |  | 11(7.7%) | 2(13%) | 1(2.4%) | 4(7.5%) | 4(12%) | Fisher's | -- | 0.280 |
| High School or Equivalent |  | 104(73%) | 10(67%) | 30(73%) | 40(75%) | 24(71%) | Fisher's | -- | 0.878 |
| Assoc/Undergraduate/Graduate Degree |  | 28(20%) | 3(20%) | 10(24%) | 9(17%) | 6(18%) | Fisher's | -- | 0.815 |
| **Lifetime Psychiatric Diagnoses** | | | | | | | | | |
| Unipolar Depression | 165 | 125(76%) | 12(67%)^ab^ | 44(85%)^a^ | 49(82%)^a^ | 20(57%)^b^ | Fisher's | -- | 0.015* |
| Bipolar Disorder | 165 | 23(14%) | 2(11%) | 7(13%) | 10(17%) | 4(11%) | Fisher's | -- | 0.920 |
| Anxiety Disorders | 165 | 55(33%) | 9(50%) | 21(40%) | 18(30%) | 7(20%) | Χ²(3) | 6.51 | 0.089 |
| Psychosis | 165 | 5(3.0%) | 1(5.6%)^a^ | 4(7.7%)^a^ | 0(0%)^b^ | 0(0%)^b^ | Fisher's | -- | 0.034* |
| PTSD | 165 | 33(20%) | 4(22%)^ab^ | 13(25%)^ab^ | 15(25%)^a^ | 1(2.9%)^b^ | Fisher's | -- | 0.017* |
| AUD | 165 | 65(39%) | 7(39%) | 23(44%) | 23(38%) | 12(34%) | Χ²(3) | 0.92 | 0.820 |
| SUD | 165 | 93(56%) | 11(61%) | 34(65%) | 31(52%) | 17(49%) | Χ²(3) | 3.29 | 0.349 |
| **Suicidality** | | | | | | | | | |
| History of Suicide Attempt | 158 | 100(63%) | 4(21%)^c^ | 19(37%)^c^ | 61(100%)^a^ | 16(62%)^b^ | Χ²(3) | 66.02 | <0.001*** |
| Family History of Suicide | 125 | 60(48%) | 9(60%)^ab^ | 20(48%)^ab^ | 28(56%)^a^ | 3(17%)^b^ | Χ²(3) | 9.23 | 0.026* |
| **Current Medication Use** | | | | | | | | | |
| Psychotropics | 117 | 108(92%) | 12(92%)^a^ | 40(98%)^a^ | 47(98%)^a^ | 9(60%)^b^ | Fisher's | -- | <0.001*** |
| Antidepressants | 120 | 93(78%) | 9(64%)^a^ | 38(93%)^a^ | 42(86%)^a^ | 4(25%)^b^ | Fisher's | -- | <0.001*** |
| Antipsychotics | 120 | 34(28%) | 6(43%) | 12(29%) | 15(31%) | 1(6.3%) | Fisher's | -- | 0.117 |
| Anticonvulsant | 120 | 23(19%) | 4(29%) | 6(15%) | 10(20%) | 3(19%) | Fisher's | -- | 0.682 |
| Antianxiety | 120 | 48(40%) | 4(29%) | 14(34%) | 24(49%) | 6(38%) | Χ²(3) | 3.04 | 0.386 |
| SUD Medications | 120 | 5(4.2%) | 0(0%) | 4(9.8%) | 1(2.0%) | 0(0%) | Fisher's | -- | 0.322 |
| Birth Control | 71 | 2(2.8%) | 0(0%) | 1(4.8%) | 0(0%) | 1(6.3%) | Fisher's | -- | 0.378 |
| **Outcome** | | | | | | | | | |
| Hair Cortisol Concentration ln(pg/mg) | 168 | 2.29(0.85) | 2.57(0.84)^a^ | 2.51(0.80)^a^ | 2.20(0.87)^ab^ | 1.95(0.77)^b^ | F(3, 164) | 4.51 | 0.006** |
| Note. *p < 0.05; **p < 0.01; ***p < 0.001. PC = psychiatric control group; SI = suicidal ideation group; SA = suicide attempt group; PTSD = posttraumatic stress disorder; AUD = alcohol use disorder; SUD = substance use disorder. | | | | | | | | | |

# Table 4. Relationships of Demographic and Clinical Characteristics to HCC Across the Spectrum of Suicidal Behaviors in the PROmiSe Sample

|  | **Beta** | **SE** | ***t*** | ***p*** |
| --- | --- | --- | --- | --- |
| **Participant Characteristics** | | | | |
| Group |  |  | *F*(3, 159) = 4.36 | 0.006** |
| SI | 0.34 | 0.13 | 2.54 | 0.012* |
| SA | −0.24 | 0.14 | −1.70 | 0.091 |
| Suicide | −0.41 | 0.17 | −2.48 | 0.014* |
| Age | 0.00 | 0.01 | 0.27 | 0.788 |
| BMI | 0.01 | 0.01 | 1.16 | 0.250 |
| Sex (Male) | 0.41 | 0.13 | 3.23 | 0.002** |
| Race (White) | −0.23 | 0.16 | −1.48 | 0.141 |
| Current Tobacco Use | 0.24 | 0.14 | 1.74 | 0.085 |
| Education |  |  | *F*(2, 135) = 0.63 | 0.534 |
| < High School | −0.27 | 0.25 | −1.07 | 0.286 |
| High School or Equivalent | 0.13 | 0.15 | 0.81 | 0.418 |
| Assoc/Undergraduate/Graduate Degree | −0.03 | 0.17 | −0.18 | 0.854 |
| **Lifetime Psychiatric Diagnoses** | | | | |
| Unipolar Depression | −0.03 | 0.15 | −0.17 | 0.863 |
| Bipolar Disorder | 0.12 | 0.19 | 0.64 | 0.524 |
| Anxiety Disorders | −0.13 | 0.14 | −0.94 | 0.347 |
| Psychosis | 0.28 | 0.38 | 0.75 | 0.457 |
| PTSD | −0.06 | 0.16 | −0.35 | 0.726 |
| AUD | 0.18 | 0.13 | 1.31 | 0.191 |
| SUD | 0.20 | 0.13 | 1.55 | 0.122 |
| **Suicide History** | | | | |
| History of Suicide Attempt | −0.22 | 0.14 | −1.65 | 0.101 |
| Family History of Suicide | 0.14 | 0.15 | 0.90 | 0.371 |
| **Medications** | | | | |
| Psychotropics | 0.25 | 0.30 | 0.84 | 0.402 |
| Antidepressants | 0.01 | 0.19 | 0.07 | 0.948 |
| Antipsychotics | −0.04 | 0.17 | −0.26 | 0.794 |
| Anticonvulsant | −0.28 | 0.19 | −1.46 | 0.148 |
| Antianxiety | −0.11 | 0.15 | −0.69 | 0.489 |
| SUD Medications | 1.36 | 0.36 | 3.81 | <0.001*** |
| Birth Control | 0.01 | 0.51 | 0.02 | 0.984 |
| Note. *p < 0.05; **p < 0.01; ***p < 0.001. HCC = hair cortisol concentration ln(pg/mg); SI = suicidal ideation group; SA = suicide attempt group; BMI = body mass index; PTSD = posttraumatic stress disorder; AUD = alcohol use disorder; SUD = substance use disorder. Analyses included HCC batch as a covariate. | | | | |

# Table 5. Pairwise Comparisons for HCC in PROmiSe Sample

| **Contrast** | **Est. diff** | ***SE*** | ***p*** | ***d*(95% CI)** |
| --- | --- | --- | --- | --- |
| SA - PC | −0.38 | 0.22 | 0.310 | 0.46(−0.07, 0.98) |
| SI - PC | −0.06 | 0.22 | 0.993 | 0.07(−0.45, 0.60) |
| SI - SA | 0.32 | 0.15 | 0.179 | 0.38(0.01, 0.76) |
| Suicide - PC | −0.63 | 0.24 | 0.042* | 0.76(0.19, 1.33) |
| Suicide - SA | −0.25 | 0.17 | 0.481 | 0.30(−0.12, 0.72) |
| Suicide - SI | −0.57 | 0.18 | 0.010* | 0.69(0.25, 1.12) |
| Note. *p < .05; **p < .01. HCC = hair cortisol concentration ln(pg/mg); PC = psychiatric control group; SI = suicidal ideation group; SA = suicide attempt group. Marginal means with Tukey-adjusted *p* values. See Figure 1A for corresponding boxplot. | | | | |

# Table 6. Sensitivity Analysis Removing Influential Points in PROmiSe and Mega-Analyses Samples

| **PROmiSe Sample (*n* = 7 removed)** | | | | | **Mega-Analyses Sample (*n* = 14 removed)** | | | | |
| --- | --- | --- | --- | --- | --- | --- | --- | --- | --- |
| **Variable** | **Beta** | **SE** | **t or F** | **p** | **Variable** | **Beta** | **SE** | ***t* or F** | ***p*** |
| Intercept | 1.87 | 0.19 | 9.83 | <0.001*** | Intercept | 2.74 | 0.26 | 10.47 | <0.001*** |
| Group (Ref = PC) |  |  | F(3, 151) = 5.77 | <0.001*** | Group (Ref = PC) |  |  | F(3, 209) = 8.63 | <0.001*** |
| SI | −0.15 | 0.20 | -0.75 | 0.456 | SI | 0.00 | 0.22 | -0.02 | 0.985 |
| SA | 0.09 | 0.19 | 0.47 | 0.642 | SA | 0.18 | 0.22 | 0.82 | 0.414 |
| Suicide | −0.54 | 0.20 | -2.68 | 0.008** | Suicide | −0.68 | 0.23 | -2.95 | 0.004** |
| Sex (Male) | 0.46 | 0.11 | 4.17 | <0.001*** | Sex (Male) | 0.33 | 0.11 | 3.09 | 0.002** |
| Batch |  |  | F(5, 151) = 5.81 | <0.001*** | Bipolar (lifetime) | 0.38 | 0.13 | 2.94 | 0.004** |
|  |  |  |  |  | SUD (lifetime) | 0.18 | 0.11 | 1.68 | 0.094 |
|  |  |  |  |  | Sample (PROmiSe) | −0.67 | 0.13 | -5.09 | <0.001*** |
|  |  |  |  |  | Batch |  |  | F(5, 209) = 14.82 | <0.001*** |
| Note. **p* < 0.05; ***p* < 0.01; ***p < 0.001. HCC = hair cortisol concentration ln(pg/mg); SI = suicidal ideation group; SA = suicide attempt group; SUD = substance use disorder. PROmiSe sample: *N* = 161, *F*(9, 151) = 6.83, *R*^2^ = 0.29, *p* < 0.001. Mega-analyses sample: *N* = 222, *F*(12, 209) = 11.49, *R*^2^ = 0.40 , *p* < 0.001. | | | | | | | | | |

# Table 7. Pairwise Comparisons for Sensitivity Model in the PROmiSe and Mega-Analyses Samples with Influential Points Removed

|  | **PROmiSe Sample (*n* = 7 removed)** | | | | **Mega-Analyses Sample (*n* = 14 removed)** | | | |
| --- | --- | --- | --- | --- | --- | --- | --- | --- |
| **Contrast** | **Est. diff** | ***SE*** | ***p*** | ***d*(95% CI)** | **Est. diff** | ***SE*** | ***p*** | ***d*(95% CI)** |
| SA - PC | −0.15 | 0.20 | 0.877 | 0.22(−0.37, 0.81) | 0.00 | 0.22 | 1.000 | 0.01(−0.58, 0.59) |
| SI - PC | 0.09 | 0.19 | 0.967 | 0.14(−0.44, 0.72) | 0.18 | 0.22 | 0.846 | 0.24(−0.34, 0.82) |
| SI - SA | 0.24 | 0.13 | 0.267 | 0.36(−0.03, 0.75) | 0.18 | 0.12 | 0.403 | 0.25(−0.07, 0.56) |
| Suicide - PC | −0.54 | 0.20 | 0.040* | 0.82(0.21, 1.42) | −0.68 | 0.23 | 0.018* | 0.92(0.30, 1.54) |
| Suicide - SA | −0.39 | 0.17 | 0.089 | 0.59(0.09, 1.09) | −0.68 | 0.18 | <0.001*** | 0.91(0.44, 1.39) |
| Suicide - SI | −0.63 | 0.16 | <0.001*** | 0.95(0.47, 1.44) | −0.86 | 0.17 | <0.001*** | 1.16(0.69, 1.63) |
| *Note.* **p* < .05; **p < 0.01; ****p* < 0.001. PC = psychiatric control group; SI = suicidal ideation group; SA = suicide attempt group. Marginal means with Tukey-adjusted p values. | | | | | | | | |

# Table 8. Sensitivity Analyses for the PROmiSe and Mega-Analyses Samples—Removing Participants with CV > 15% (*n* = 5)

| **PROmiSe Sample** | | | | | **Mega-Analyses Sample** | | | | |
| --- | --- | --- | --- | --- | --- | --- | --- | --- | --- |
| **Variable** | **Beta** | ***SE*** | ***t*** | ***p*** | **Variable** | **Beta** | ***SE*** | ***t*** | ***p*** |
| Intercept | 1.99 | 0.21 | 9.62 | <0.001*** | Intercept | 2.78 | 0.29 | 9.5 | <0.001*** |
| Group (Ref = PC) |  |  | F(3, 152) = 4.49 | 0.005** | Group (Ref = PC) |  |  | F(3, 216) = 6.33 | <0.001*** |
| SI | −0.29 | 0.21 | -1.36 | 0.177 | SI | −0.08 | 0.25 | -0.35 | 0.730 |
| SA | −0.01 | 0.21 | -0.07 | 0.946 | SA | 0.14 | 0.24 | 0.6 | 0.551 |
| Suicide | −0.60 | 0.22 | -2.7 | 0.008** | Suicide | −0.71 | 0.26 | -2.69 | 0.008** |
| Sex (Male) | 0.45 | 0.13 | 3.56 | <0.001*** | Sex (Male) | 0.30 | 0.13 | 2.37 | 0.019* |
| Batch |  |  | F(5, 152) = 4.29 | 0.001** | Bipolar (lifetime) | 0.32 | 0.15 | 2.18 | 0.030* |
|  |  |  |  |  | SUD (lifetime) | 0.19 | 0.13 | 1.5 | 0.135 |
|  |  |  |  |  | Sample (PROmiSe) | −0.57 | 0.15 | -3.79 | <0.001*** |
|  |  |  |  |  | Batch |  |  | F(5, 216) = 7.41 | <0.001*** |
| Note. **p* < 0.05; ***p* < 0.01; ***p < 0.001. HCC = hair cortisol concentration ln(pg/mg); CV = coefficient of variation; SI = suicidal ideation group; SA = suicide attempt group; SUD = substance use disorder. PROmiSe sample: *N* = 162, *F*(9, 152) = 5.30, *R*^2^ = 0.24, *p* < 0.001. Mega-analyses sample: *N* = 229, *F*(12, 216) = 6.71, *R*^2^ = 0.26, *p* < 0.001. | | | | | | | | | |

# Table 9. Pairwise Comparisons for PROmiSe and Mega-Analyses Final Multivariate Models Removing Participants with CVs > 15% (*n* = 5)

|  | **PROmiSe Sample** | | | | **Mega-Analyses Sample** | | | |
| --- | --- | --- | --- | --- | --- | --- | --- | --- |
| **Contrast** | **Est. diff** | ***SE*** | ***p*** | ***d*(95% CI)** | **Est. diff** | ***SE*** | ***p*** | ***d*(95% CI)** |
| SA - PC | −0.29 | 0.21 | 0.528 | 0.39(−0.18, 0.95) | −0.08 | 0.25 | 0.986 | 0.10(−0.46, 0.65) |
| SI - PC | −0.01 | 0.21 | 1.000 | 0.02(−0.53, 0.57) | 0.14 | 0.24 | 0.933 | 0.17(−0.38, 0.71) |
| SI - SA | 0.28 | 0.15 | 0.244 | 0.37(−0.02, 0.76) | 0.23 | 0.13 | 0.322 | 0.26(−0.04, 0.57) |
| Suicide - PC | −0.60 | 0.22 | 0.039* | 0.80(0.21, 1.39) | −0.71 | 0.26 | 0.038* | 0.81(0.21, 1.42) |
| Suicide - SA | −0.31 | 0.19 | 0.364 | 0.41(−0.09, 0.91) | −0.63 | 0.21 | 0.015* | 0.72(0.25, 1.19) |
| Suicide - SI | −0.58 | 0.18 | 0.008** | 0.78(0.30, 1.26) | −0.86 | 0.20 | <0.001*** | 0.98(0.52, 1.44) |
| Note. *p < .05; **p < .01. HCC = hair cortisol concentration ln(pg/mg); PC = psychiatric control group; SI = suicidal ideation group; SA = suicide attempt group. Marginal means with Tukey-adjusted *p* values. | | | | | | | | |

# Table 10. Sensitivity Analyses for the PROmiSe and Mega-Analyses Samples—PC (*n* = 4) and SI (*n* = 19) Participants with History of Suicide Attempt Removed

| **PROmiSe Sample** | | | | | **Mega-Analyses Sample** | | | | |
| --- | --- | --- | --- | --- | --- | --- | --- | --- | --- |
| **Variable** | **Beta** | ***SE*** | ***t*** | ***p*** | **Variable** | **Beta** | ***SE*** | ***t*** | ***p*** |
| Intercept | 1.97 | 0.23 | 8.48 | <0.001*** | Intercept | 2.79 | 0.31 | 8.97 | <0.001*** |
| Group |  |  | F(3, 135) = 4.78 | 0.003** | Group |  |  | F(3, 200) = 7.1 | <0.001*** |
| Group: SA | −0.27 | 0.24 | -1.14 | 0.258 | Group: SA | −0.07 | 0.26 | -0.27 | 0.786 |
| Group: SI | 0.08 | 0.25 | 0.31 | 0.753 | Group: SI | 0.21 | 0.27 | 0.77 | 0.444 |
| Group: Suicide | −0.62 | 0.24 | -2.54 | 0.012* | Group: Suicide | −0.76 | 0.28 | -2.73 | 0.007** |
| Sex (Male) | 0.43 | 0.14 | 3.15 | 0.002** | Sex (Male) | 0.27 | 0.13 | 2.05 | 0.041* |
| Batch |  |  | F(5, 135) = 3.55 | 0.005** | Bipolar | 0.34 | 0.16 | 2.19 | 0.030* |
|  |  |  |  |  | SUD | 0.20 | 0.13 | 1.54 | 0.125 |
|  |  |  |  |  | sample | −0.58 | 0.16 | -3.65 | <0.001*** |
|  |  |  |  |  | Batch |  |  | F(5, 200) = 8.19 | <0.001*** |
| Note. **p* < 0.05; ***p* < 0.01; ***p < 0.001. HCC = hair cortisol concentration ln(pg/mg); CV = coefficient of variation; SI = suicidal ideation group; SA = suicide attempt group; SUD = substance use disorder. PROmiSe sample: *N* = 145, *F*(9, 135) = 4.46, *R*^2^ = 0.23, *p* < 0.001. Mega-analyses sample: *N* = 213, *F*(12, 200) = 7.15, *R*^2^ = 0.30, *p* < 0.001. | | | | | | | | | |

# Table 11. Pairwise Comparisons for PROmiSe and Mega-Analyses Final Multivariate Models Removing PC (*n =* 4) and SI (*n* = 19) Participants with History of Suicide Attempt

|  | **PROmiSe Sample** | | | | **Mega-Analyses Sample** | | | |
| --- | --- | --- | --- | --- | --- | --- | --- | --- |
| **Contrast** | **Est. diff** | ***SE*** | ***p*** | ***d*(95% CI)** | **Est. diff** | ***SE*** | ***p*** | ***d*(95% CI)** |
| SA - PC | −0.27 | 0.24 | 0.668 | 0.35(−0.26, 0.95) | −0.07 | 0.26 | 0.993 | 0.08(−0.51, 0.67) |
| SI - PC | 0.08 | 0.25 | 0.989 | 0.10(−0.53, 0.73) | 0.21 | 0.27 | 0.869 | 0.23(−0.37, 0.83) |
| SI - SA | 0.35 | 0.17 | 0.181 | 0.45(0.01, 0.89) | 0.28 | 0.14 | 0.229 | 0.31(−0.01, 0.64) |
| Suicide - PC | −0.62 | 0.24 | 0.058 | 0.80(0.17, 1.44) | −0.76 | 0.28 | 0.034* | 0.86(0.24, 1.49) |
| Suicide - SA | −0.35 | 0.20 | 0.282 | 0.46(−0.05, 0.96) | −0.69 | 0.21 | 0.006** | 0.78(0.31, 1.26) |
| Suicide - SI | −0.70 | 0.20 | 0.004** | 0.90(0.38, 1.43) | −0.96 | 0.21 | <0.001*** | 1.10(0.61, 1.59) |
| Note. *p < .05; **p < .01. HCC = hair cortisol concentration ln(pg/mg); PC = psychiatric control group; SI = suicidal ideation group; SA = suicide attempt group. Marginal means with Tukey-adjusted *p* values. | | | | | | | | |

# Table 12. Final Multivariate Regression Models for the PROmiSe and Mega-Analyses Samples—Participants Taking SUD Medications Removed (*n* = 5)

| **PROmiSe Sample** | | | | | **Mega-Analyses Sample** | | | | |
| --- | --- | --- | --- | --- | --- | --- | --- | --- | --- |
| **Variable** | **Beta** | ***SE*** | ***t*** | ***p*** | **Variable** | **Beta** | ***SE*** | ***t*** | ***p*** |
| Intercept | 2.01 | 0.20 | 10 | <0.001*** | Intercept | 2.92 | 0.28 | 10.31 | <0.001*** |
| Group (Ref = PC) |  |  | F(3, 153) = 4.98 | 0.003** | Group (Ref = PC) |  |  | F(3, 217) = 6.82 | <0.001*** |
| SI | −0.32 | 0.20 | -1.56 | 0.120 | SI | −0.15 | 0.23 | -0.66 | 0.507 |
| SA | −0.02 | 0.20 | -0.1 | 0.919 | SA | 0.09 | 0.23 | 0.41 | 0.683 |
| Suicide | −0.61 | 0.21 | -2.89 | 0.004** | Suicide | −0.75 | 0.25 | -3 | 0.003** |
| Sex (Male) | 0.39 | 0.12 | 3.22 | 0.002** | Sex (Male) | 0.23 | 0.12 | 1.88 | 0.061 |
| Batch |  |  | F(5, 153) = 5.26 | <0.001*** | Bipolar (lifetime) | 0.31 | 0.15 | 2.1 | 0.037* |
|  |  |  |  |  | SUD (lifetime) | 0.19 | 0.12 | 1.61 | 0.110 |
|  |  |  |  |  | Sample (PROmiSe) | −0.67 | 0.15 | -4.5 | <0.001*** |
|  |  |  |  |  | Batch |  |  | F(5, 217) = 9.77 | <0.001*** |
| Note. **p* < 0.05; ***p* < 0.01; ***p < 0.001. HCC = hair cortisol concentration ln(pg/mg); SI = suicidal ideation group; SA = suicide attempt group; SUD = substance use disorder. PROmiSe sample: *N* = 165, *F*(9, 155) = 5.59, *R*^2^ = 0.25, *p* < 0.001. Mega-analyses sample: *N* = 2229, *F*(12, 216) = 7.56, *R*^2^ = 0.30, *p* < 0.001. | | | | | | | | | |

# Table 13. Post-Hoc Pairwise Comparisons in Final Models for the PROmiSe and Mega-Analyses Samples with Participants Taking SUD Medications Removed (*n* = 5)

|  | **PROmiSe Sample** | | | | **Mega-Analyses Sample** | | | |
| --- | --- | --- | --- | --- | --- | --- | --- | --- |
| **Contrast** | **Est. diff** | ***SE*** | ***p*** | ***d*(95% CI)** | **Est. diff** | ***SE*** | ***p*** | ***d*(95% CI)** |
| SA - PC | −0.32 | 0.20 | 0.403 | 0.43(−0.12, 0.98) | −0.15 | 0.23 | 0.910 | 0.18(−0.36, 0.72) |
| SI - PC | −0.02 | 0.20 | 1.000 | 0.03(−0.52, 0.57) | 0.09 | 0.23 | 0.977 | 0.11(−0.43, 0.65) |
| SI - SA | 0.30 | 0.14 | 0.174 | 0.41(0.01, 0.80) | 0.25 | 0.13 | 0.232 | 0.29(−0.01, 0.60) |
| Suicide - PC | −0.61 | 0.21 | 0.023* | 0.84(0.26, 1.42) | −0.75 | 0.25 | 0.016* | 0.89(0.30, 1.48) |
| Suicide - SA | −0.30 | 0.18 | 0.362 | 0.41(−0.09, 0.90) | −0.60 | 0.20 | 0.014* | 0.71(0.24, 1.17) |
| Suicide - SI | −0.59 | 0.18 | 0.006** | 0.81(0.32, 1.30) | −0.85 | 0.19 | <0.001*** | 1.00(0.54, 1.47) |
| Note. *p < .05; **p < .01. HCC = hair cortisol concentration ln(pg/mg); PC = psychiatric control group; SI = suicidal ideation group; SA = suicide attempt group. Marginal means with Tukey-adjusted *p* values. | | | | | | | | |

# Table 14. Sample Characteristics and Group Comparisons in Mega-Analysis Sample

|  |  | **Mean(*SD*) or *n*(%)** | | | | |  |  |  |
| --- | --- | --- | --- | --- | --- | --- | --- | --- | --- |
|  | ***N*** | **Overall**  *N* = 238 | **PC**  *n* = 19 | **SI**  *n* = 88 | **SA**  *n* = 96 | **Suicide**  N = 35 | **Test** | **Statistic** | ***p*** |
| **Participant Characteristics** | | | | | | | | | |
| Age | 238 | 24.25(4.68) | 23.04(3.46)^b^ | 24.24(3.97)^b^ | 23.34(3.92)^b^ | 27.40(7.03)^a^ | F(3, 234) | 4.07 | 0.010* |
| BMI | 213 | 25.37(5.51) | 25.83(5.23) | 25.22(5.12) | 25.90(6.14) | 24.15(4.81) | F(3, 209) | 0.92 | 0.437 |
| Sex (Male) | 238 | 135(57%) | 13(68%)^ab^ | 52(59%)^ab^ | 43(45%)^b^ | 27(77%)^a^ | Χ²(3) | 12.77 | 0.005** |
| Race (White) | 237 | 189(80%) | 14(74%) | 68(77%) | 74(78%) | 33(94%) | Fisher's | -- | 0.096 |
| **Lifetime Psychiatric Diagnoses** | | | | | | | | | |
| Unipolar Depression | 235 | 165(70%) | 12(67%) | 67(77%) | 66(69%) | 20(57%) | Χ²(3) | 4.91 | 0.178 |
| Bipolar Disorder | 235 | 49(21%) | 2(11%) | 18(21%) | 25(26%) | 4(11%) | Fisher's | -- | 0.228 |
| Anxiety Disorders | 235 | 102(43%) | 9(50%)^ab^ | 43(49%)^a^ | 43(45%)^a^ | 7(20%)^b^ | Χ²(3) | 9.54 | 0.023* |
| Psychosis | 235 | 7(3.0%) | 1(5.6%)^ab^ | 6(6.9%)^a^ | 0(0%)^b^ | 0(0%)^ab^ | Fisher's | -- | 0.016* |
| PTSD | 235 | 90(38%) | 4(22%)^ab^ | 43(49%)^a^ | 42(44%)^a^ | 1(2.9%)^b^ | Χ²(3) | 26.54 | <0.001*** |
| AUD | 235 | 104(44%) | 7(39%) | 40(46%) | 45(47%) | 12(34%) | Χ²(3) | 2.10 | 0.552 |
| SUD | 235 | 143(61%) | 11(61%) | 58(67%) | 57(60%) | 17(49%) | Χ²(3) | 3.48 | 0.32 |
| **Suicidality** | | | | | | | | | |
| History of Suicide Attempt | 158 | 100(63%) | 4(21%)^c^ | 19(37%)^c^ | 61(100%)^a^ | 16(62%)^b^ | Χ²(3) | 66.02 | <0.001*** |
| Family History of Suicide | 125 | 60(48%) | 9(60%)^ab^ | 20(48%)^ab^ | 28(56%)^a^ | 3(17%)^b^ | Χ²(3) | 9.23 | 0.026* |
| **Current Medication Use** | | | | | | | | | |
| Psychotropics | 158 | 146(92%) | 12(92%)^a^ | 57(93%)^a^ | 68(99%)^a^ | 9(60%)^b^ | Fisher's | -- | <0.001*** |
| Antidepressants | 161 | 124(77%) | 9(64%)^a^ | 52(85%)^a^ | 59(84%)^a^ | 4(25%)^b^ | Fisher's | -- | <0.001*** |
| Antipsychotics | 161 | 41(25%) | 6(43%) | 15(25%) | 19(27%) | 1(6.3%) | Fisher's | -- | 0.131 |
| Anticonvulsant | 161 | 27(17%) | 4(29%) | 7(11%) | 13(19%) | 3(19%) | Fisher's | -- | 0.378 |
| Antianxiety | 161 | 60(37%) | 4(29%) | 19(31%) | 31(44%) | 6(38%) | Χ²(3) | 2.91 | 0.406 |
| SUD Medications | 161 | 8(5.0%) | 0(0%) | 5(8.2%) | 3(4.3%) | 0(0%) | Fisher's | -- | 0.581 |
| Birth Control | 112 | 7(6.3%) | 0(0%) | 3(7.3%) | 3(5.9%) | 1(6.3%) | Fisher's | -- | 1.000 |
| **Outcome** | | | | | | | | | |
| Hair Cortisol Concentration ln(pg/mg) | 238 | 2.46(1.00) | 2.57(0.84)^ab^ | 2.70(1.00)^a^ | 2.40(1.04)^ab^ | 1.95(0.77)^b^ | F(3, 234) | 6.72 | <0.001*** |
| *Note.* *p<0.05; **p<0.01; ***p<0.001. PC = psychiatric control group; SI = suicidal ideation group; SA = suicide attempt group; PTSD = posttraumatic stress disorder; AUD = alcohol use disorder; SUD = substance use disorder. Lettered superscripts denote significant group differences. | | | | | | | | | |

# Table 15. Relationships of Demographic and Clinical Characteristics to HCC in Mega-Analyses Sample

|  | **Beta** | ***SE*** | ***t*** | ***p*** |
| --- | --- | --- | --- | --- |
| **Participant Characteristics** | | | | |
| Group (Ref = PC) |  |  | *F*(3, 229) = 9.78 | <0.001*** |
| SA | 0.45 | 0.13 | 3.44 | <0.001*** |
| SI | −0.02 | 0.14 | −0.16 | 0.872 |
| Suicide | −0.93 | 0.19 | −4.95 | <0.001*** |
| Age | −0.01 | 0.01 | −0.97 | 0.333 |
| BMI | 0.01 | 0.01 | 0.90 | 0.372 |
| Sex (Male) | 0.29 | 0.13 | 2.21 | 0.028* |
| Race (White) | −0.16 | 0.16 | −1.00 | 0.316 |
| **Lifetime Psychiatric Diagnoses** | | | | |
| Unipolar Depression | −0.23 | 0.14 | −1.58 | 0.114 |
| Bipolar Disorder | 0.49 | 0.16 | 3.09 | 0.002** |
| Anxiety Disorders | 0.18 | 0.14 | 1.34 | 0.182 |
| Psychosis | −0.49 | 0.38 | −1.28 | 0.201 |
| PTSD | 0.56 | 0.14 | 4.03 | <0.001*** |
| AUD | 0.25 | 0.13 | 1.89 | 0.060 |
| SUD | 0.43 | 0.13 | 3.18 | 0.002** |
| **Suicidality** | | | | |
| History of Suicide Attempt | −0.22 | 0.14 | −1.65 | 0.101 |
| Family History of Suicide | 0.14 | 0.15 | 0.90 | 0.371 |
| **Current Medication Use** | | | | |
| Psychotropics | 0.54 | 0.30 | 1.81 | 0.072 |
| Antidepressants | 0.13 | 0.19 | 0.69 | 0.488 |
| Antipsychotics | 0.05 | 0.18 | 0.26 | 0.796 |
| Anticonvulsant | −0.21 | 0.21 | −1.02 | 0.307 |
| Antianxiety | 0.07 | 0.16 | 0.41 | 0.684 |
| SUD Medications | 1.30 | 0.34 | 3.80 | <0.001*** |
| Birth Control | 0.21 | 0.38 | 0.54 | 0.590 |
| *Note.* **p* < .05; ***p* < .01; ****p* < .001. HCC = hair cortisol concentration ln(pg/mg); PC = psychiatric control group; SI = suicidal ideation group; SA = suicide attempt group; PTSD = posttraumatic stress disorder; AUD = alcohol use disorder; SUD = substance use disorder. Linear regression models controlled for HCC batch effects. | | | | |

# Table 16. Pairwise Comparisons for HCC in Mega-Analyses Sample

| **Contrast** | **Est. diff** | ***SE*** | ***p*** | ***d*(95% CI)** |
| --- | --- | --- | --- | --- |
| SA - PC | −0.17 | 0.24 | 0.896 | 0.18(−0.32, 0.67) |
| SI - PC | 0.12 | 0.25 | 0.957 | 0.13(−0.37, 0.63) |
| SI - SA | 0.30 | 0.14 | 0.168 | 0.30(0.01, 0.60) |
| Suicide - PC | −0.63 | 0.28 | 0.111 | 0.64(0.08, 1.21) |
| Suicide - SA | −0.45 | 0.19 | 0.086 | 0.47(0.08, 0.86) |
| Suicide - SI | −0.75 | 0.19 | <0.001*** | 0.77(0.37, 1.17) |
| *Note.* **p* < .05; ***p* < .01; ****p* < .001. HCC = hair cortisol concentration ln(pg/mg); PC = psychiatric control group; SI = suicidal ideation group; SA = suicide attempt group. Marginal means with Tukey-adjusted *p* values. See Figure 1B for corresponding boxplot. | | | | |

# Table 17. Sample Characteristics and Group Comparisons in the Postmortem Sample

|  |  | **Mean(*SD*) or *n*(%)** | | |  |  |  |  |
| --- | --- | --- | --- | --- | --- | --- | --- | --- |
|  | ***N*** | **Overall**  *N* = 78 | **Overdose**  *n* = 43 | **Suicide**  *n* = 35 | **Test** | **Statistic** | ***p*** | ***d*(95% CI)** |
| **Participant Characteristics** | | | | | | | | |
| Age | 78 | 29.87(6.45) | 31.88(5.20) | 27.40(7.03) | *t*(61.2) | 3.14 | 0.003** | 0.74(0.27, 1.20) |
| BMI | 76 | 25.73(5.53) | 26.93(5.79) | 24.15(4.81) | *t*(73.5) | 2.28 | 0.025* | 0.52(0.05, 0.98) |
| Sex (Male) | 78 | 51(65%) | 24(56%) | 27(77%) | χ^2^(1) | 3.88 | 0.049* | 0.45(-0.01, 0.91) |
| Race (White) | 78 | 74(95%) | 41(95%) | 33(94%) | Fisher’s | -- | >0.999 | 0.05(-0.41, 0.50) |
| Hollingshead SES | 65 | 33.45(13.54) | 31.66(12.88) | 35.53(14.20) | *t*(59.2) | -1.14 | 0.257 | 0.29(-0.21, 0.79) |
| LGBT | 78 | 45(58%) | 21(49%) | 24(69%) | χ^2^(1) | 3.08 | 0.079 | 0.40(-0.06, 0.86) |
| Veteran | 65 | 4(6.2%) | 2(6.1%) | 2(6.3%) | Fisher’s | -- | >0.999 | 0.01(-0.49, 0.50) |
| Strauss-Carpenter Outcome Scale Total Score | 43 | 10.16(3.36) | 8.55(3.17) | 11.57(2.90) | *t*(38.9) | -3.23 | 0.002** | 0.99(0.34, 1.65) |
| Current Tobacco Use | 46 | 30(65%) | 20(100%) | 10(38%) | χ^2^(1) | 18.9 | <0.001*** | 1.65(0.95, 2.34) |
| Education | 74 |  |  |  | Fisher’s | -- | 0.929 |  |
| < High School | 74 | 9(12%) | 5(13%) | 4(12%) | Fisher’s | -- | >0.999 | 0.02(-0.44, 0.49) |
| High School or Equivalent | 74 | 54(73%) | 30(75%) | 24(71%) | χ^2^(1) | 0.181 | 0.670 | 0.10(-0.37, 0.56) |
| Assoc/Undergraduate/Graduate Degree | 74 | 11(15%) | 5(13%) | 6(18%) | χ^2^(1) | 0.385 | 0.535 | 0.14(-0.32, 0.61) |
| **Lifetime Psychiatric Diagnoses** | | | | | | | | |
| Unipolar Depression | 78 | 31(40%) | 11(26%) | 20(57%) | χ^2^(1) | 8.03 | 0.005** | 0.67(0.21, 1.14) |
| Bipolar Disorder | 78 | 5(6.4%) | 1(2.3%) | 4(11%) | Fisher’s | -- | 0.168 | 0.37(-0.08, 0.83) |
| Anxiety Disorders | 78 | 16(21%) | 9(21%) | 7(20%) | χ^2^(1) | 0.010 | 0.919 | 0.02(-0.43, 0.48) |
| PTSD | 78 | 4(5.1%) | 3(7.0%) | 1(2.9%) | Fisher’s | -- | 0.623 | 0.19(-0.27, 0.64) |
| AUD | 78 | 33(42%) | 21(49%) | 12(34%) | χ^2^(1) | 1.67 | 0.196 | 0.29(-0.16, 0.75) |
| SUD | 78 | 60(77%) | 43(100%) | 17(49%) | χ^2^(1) | 28.7 | <0.001*** | 1.52(1.00, 2.03) |
| **Suicide History** | | | | | | | | |
| History of Suicide Attempt | 45 | 18(40%) | 2(11%) | 16(62%) | χ^2^(1) | 11.9 | <0.001*** | 1.19(0.53, 1.85) |
| Family History of Suicide | 38 | 7(18%) | 4(20%) | 3(17%) | Fisher’s | -- | >0.999 | 0.08(-0.58, 0.74) |
| **Life Stressors Prior to Death** | | | | | | | | |
| Interpersonal Factors | 78 | 13(17%) | 0(0%) | 13(37%) | χ^2^(1) | 19.2 | <0.001*** | 1.13(0.64, 1.62) |
| Non-interpersonal Factors | 78 | 18(23%) | 1(2.3%) | 17(49%) | χ^2^(1) | 23.2 | <0.001*** | 1.29(0.79, 1.79) |
| Both | 78 | 10(13%) | 0(0%) | 10(29%) | Fisher’s | -- | <0.001*** | 0.93(0.45, 1.41) |
| **Other Medical Conditions** | | | | | | | | |
| Seizure Disorder (lifetime) | 77 | 8(10%) | 5(12%) | 3(8.8%) | Fisher’s | -- | >0.999 | 0.09(-0.37, 0.55) |
| Head Injury | 78 | 12(15%) | 6(14%) | 6(17%) | χ^2^(1) | 0.151 | 0.698 | 0.09(-0.37, 0.54) |
| Hypertension | 77 | 3(3.9%) | 3(7.0%) | 0(0%) | Fisher’s | -- | 0.251 | 0.36(-0.10, 0.82) |
| **Medications at Time of Death** | | | | | | | | |
| Psychotropics | 31 | 22(71%) | 13(81%) | 9(60%) | Fisher’s | -- | 0.252 | 0.47(-0.28, 1.21) |
| Antidepressants | 32 | 13(41%) | 9(56%) | 4(25%) | χ^2^(1) | 3.24 | 0.072 | 0.65(-0.09, 1.39) |
| Antipsychotics | 32 | 3(9.4%) | 2(13%) | 1(6.3%) | Fisher’s | -- | >0.999 | 0.21(-0.52, 0.93) |
| Anticonvulsant | 32 | 5(16%) | 2(13%) | 3(19%) | Fisher’s | -- | >0.999 | 0.17(-0.56, 0.89) |
| Antianxiety | 32 | 12(38%) | 6(38%) | 6(38%) | χ^2^(1) | 0.000 | >0.999 | 0.00(-0.89, 0.89) |
| SUD Medications | 32 | 1(3.1%) | 1(6.3%) | 0(0%) | Fisher’s | -- | >0.999 | 0.35(-0.37, 1.08) |
| Birth Control | 32 | 1(3.1%) | 0(0%) | 1(6.3%) | Fisher’s | -- | >0.999 | 0.35(-0.37, 1.08) |
| **Outcome** | | | | | | | | |
| Hair Cortisol Concentration ln(pg/mg) | 78 | 2.20(1.08) | 2.41(1.25) | 1.95(0.77) | *t*(71.4) | 2.00 | 0.050* | 0.43(-0.02, 0.89) |
| *Note.* *p<0.05; **p<0.01; ***p<0.001. BMI = body mass index; SES = socioeconomic status; PTSD = posttraumatic stress disorder; AUD = alcohol use disorder; SUD = substance use disorder; Seizure Disorder = lifetime history of seizure, including psychogenic nonepileptic seizures. | | | | | | | | |

# Table 18. Relationships of Demographic and Clinical Characteristics to HCC in the Postmortem Sample

|  | **Beta** | ***SE*** | ***t*** | ***p*** |
| --- | --- | --- | --- | --- |
| **Participant Characteristics** | | | | |
| Age | 0.02 | 0.02 | 1.14 | 0.259 |
| BMI | 0.01 | 0.02 | 0.32 | 0.751 |
| Sex (Male) | -0.29 | 0.24 | -1.24 | 0.218 |
| Race (White) | -0.27 | 0.51 | -0.53 | 0.599 |
| Hollingshead SES | 0.01 | 0.01 | 0.55 | 0.585 |
| LGBT | -0.25 | 0.23 | -1.06 | 0.291 |
| Veteran | 0.45 | 0.49 | 0.92 | 0.361 |
| Strauss-Carpenter Outcome Scale Total Score | -0.04 | 0.05 | -0.85 | 0.401 |
| Current Tobacco Use | 0.57 | 0.33 | 1.73 | 0.090 |
| Education |  |  | *F*(2, 69) = 0.01 | 0.990 |
| < High School | 0.03 | 0.35 | 0.1 | 0.923 |
| High School or Equivalent | -0.04 | 0.26 | -0.15 | 0.885 |
| Assoc/Undergraduate/Graduate Degree | 0.03 | 0.33 | 0.09 | 0.926 |
| **Lifetime Psychiatric Diagnoses** | | | | |
| Unipolar Depression | -0.31 | 0.23 | -1.36 | 0.179 |
| Bipolar Disorder | -0.11 | 0.46 | -0.24 | 0.807 |
| Anxiety Disorders | -0.25 | 0.28 | -0.86 | 0.391 |
| Lifetime PTSD | 0.41 | 0.52 | 0.80 | 0.428 |
| Lifetime AUD | 0.35 | 0.23 | 1.53 | 0.131 |
| Lifetime SUD | 0.23 | 0.27 | 0.85 | 0.398 |
| **Suicide History** | | | | |
| History of Suicide Attempt | -0.05 | 0.34 | -0.15 | 0.879 |
| Family History of Suicide | -0.10 | 0.49 | -0.2 | 0.840 |
| **Factors Associated with the Death** | | | | |
| Interpersonal Factors | -0.18 | 0.30 | -0.58 | 0.566 |
| Non-interpersonal Factors | -0.21 | 0.27 | -0.79 | 0.429 |
| Both | -0.36 | 0.34 | -1.04 | 0.301 |
| **Other Medical Conditions** | | | | |
| Seizure Disorder (lifetime) | 1.01 | 0.37 | 2.75 | 0.008** |
| Head Injury | 0.40 | 0.32 | 1.23 | 0.221 |
| Hypertension | 0.90 | 0.59 | 1.54 | 0.127 |
| **Medications at Time of Death** | | | | |
| Psychotropics | 0.24 | 0.28 | 0.88 | 0.386 |
| Antidepressants | -0.10 | 0.25 | -0.40 | 0.695 |
| Antipsychotics | -0.05 | 0.42 | -0.11 | 0.911 |
| Anticonvulsant | 0.40 | 0.34 | 1.2 | 0.242 |
| Antianxiety | 0.30 | 0.25 | 1.23 | 0.229 |
| SUD Medications | 0.71 | 0.73 | 0.97 | 0.339 |
| Birth Control | -0.01 | 0.69 | -0.01 | 0.992 |
| *Note.* *p < .05; **p < .01; ***p < .001. HCC = hair cortisol concentration ln(pg/mg); BMI = body mass index; SES = socioeconomic status; LGBT = lesbian, gay, bisexual, and trans identity; PTSD = posttraumatic stress disorder; AUD = alcohol use disorder; SUD = substance use disorder. Linear regression models controlled for HCC batch effects. | | | | |

# Table 19. Sensitivity Analysis in the Postmortem Sample with Influential Points Removed (*n* = 3)

| **Variable** | **Beta** | **SE** | ***t*** | ***p*** |
| --- | --- | --- | --- | --- |
| Intercept | 1.93 | 0.16 | 12.45 | <0.001*** |
| Group: Suicide | −0.39 | 0.19 | -2.1 | 0.040* |
| Seizure Disorder (lifetime) | 0.66 | 0.35 | 1.88 | 0.065 |
| Batch |  |  | *F*(2, 69) = 11.09 | <0.001*** |
| Note. *p < .05; **p < .01; ***p < .001. *N* = 74, *F*(4, 69) = 8.83, *R*^2^ = 0.34, *p* = < 0.001. Seizure Disorder = lifetime history of seizure, including psychogenic nonepileptic seizures. Number of influential points removed = 3. Group Cohen's *d*(95% CI) = 0.04(-0.89, 0.80). | | | | |

# Table 20. Sensitivity Analyses for the Postmortem Sample—Removing Participants with CV > 15% (*n* = 1)

| **Variable** | **Beta** | ***SE*** | ***t*** | ***p*** |
| --- | --- | --- | --- | --- |
| Intercept | 1.90 | 0.18 | 10.66 | <0.001*** |
| Group: Suicide | −0.34 | 0.22 | -1.58 | 0.120 |
| Seizure Disorder (lifetime) | 0.98 | 0.36 | 2.72 | 0.008** |
| Batch |  |  | *F*(2, 71) = 7.72 | 0.001** |
| Note. **p* < 0.05; ***p* < 0.01; ***p < 0.001. HCC = hair cortisol concentration ln(pg/mg); Seizure Disorder = lifetime history of seizure, including psychogenic nonepileptic seizures. *N* = 76, *F*(4, 71) = 6.85, *R*^2^ = 0.28, *p* < 0.001. Suicide-Overdose contrast effect size: *d* = 0.4, 95% CI [-0.06, 0.87]. | | | | |

# Table 21. Sensitivity Analyses for the Postmortem Sample—Removing Participant with Long Postmortem Interval Time

| **Variable** | **Beta** | ***SE*** | ***t*** | ***p*** |
| --- | --- | --- | --- | --- |
| Intercept | 1.80 | 0.17 | 10.31 | <0.001*** |
| Group: Suicide | −0.31 | 0.21 | −1.50 | 0.137 |
| Seizure Disorder (lifetime) | 1.04 | 0.35 | 2.98 | 0.004** |
| Batch |  |  | F(2, 71) = 10.10 | <0.001 *** |
| Note. **p* < 0.05; ***p* < 0.01; ***p < 0.001. HCC = hair cortisol concentration ln(pg/mg); Seizure Disorder = lifetime history of seizure, including psychogenic nonepileptic seizures. *N* = 76, *F*(4, 71) = 8.54, *R*^2^ = 0.32, *p* < 0.001. Suicide-Overdose contrast effect size: *d* = 0.35, 95% CI [-0.12, 0.82]. | | | | |


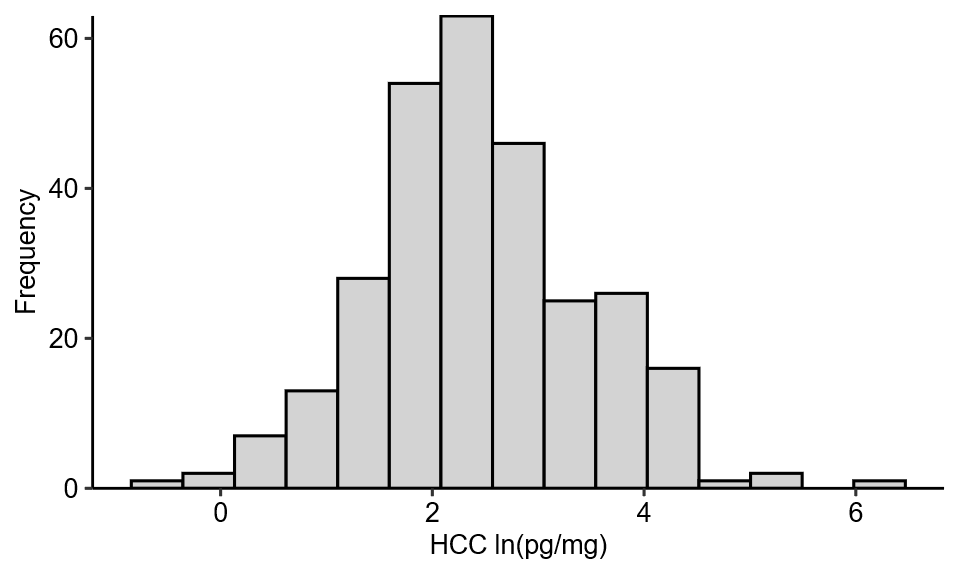


# Figure 1. Distribution of log-transformed hair cortisol concentration data
